# Muscle Union Procedure in Patients with Paralytic Strabismus

**DOI:** 10.1371/journal.pone.0129035

**Published:** 2015-06-12

**Authors:** Kyung-Ah Park, Injeong Lyu, Jungmin Yoon, Unchang Jeong, Jae-Eung Oh, Han Woong Lim, Sei Yeul Oh

**Affiliations:** 1 Department of Ophthalmology, Samsung Medical Center, Sungkyunkwan University School of Medicine, Seoul, Korea; 2 Hanyang University School of Mechanical Engineering, Seoul, Korea; 3 Department of Ophthalmology, College of Medicine, Hanyang University, Seoul, Korea; Medical College of Soochow University, CHINA

## Abstract

To present the surgical outcomes of a muscle union procedure in patients with paralytic strabismus, this retrospective study included 27 patients with paralytic strabismus who underwent a muscle union procedure. In this procedure, the two vertical rectus muscles are united with the paralytic horizontal muscle without splitting the muscles. Postoperative ocular deviations, complications, surgical success rates, and reoperation rates were obtained by examining the medical records of the patients. Seventeen patients had a sixth cranial nerve palsy, seven patients had a third cranial nerve palsy, and three patients had a medial rectus muscle palsy after endoscopic sinus surgery. The mean preoperative angle of horizontal deviation in the primary position was 56 ± 21 prism diopters. The mean follow-up period was 12 ± 9 months. The mean final postoperative ocular deviation was 8 ± 13 prism diopters. The success rate was 74%, and the reoperation rate was 0%. No significant complications, including anterior ischemia, occurred in any of the patients. One patient exhibited an increase in intraocular pressure in the immediate postoperative period, but this resolved spontaneously within 1 week. Our muscle union procedure was effective in patients with paralytic strabismus, especially in patients with a large angle of deviation. This muscle union procedure is potentially a suitable option for muscle transposition in patients with paralytic strabismus who have large-angle deviation or a significant residual angle after conventional surgery.

## Introduction

The most effective surgical method available for large-angle strabismus with minimal or absent muscle force in paralytic strabismus is full tendon transposition augmented with posterior fixation [1–3] or posterior intermuscular suture [4]. In these approaches, anterior segment ischemia is a rare, but potentially serious complication. The anterior ciliary vessels of the vertical rectus muscles are believed to play a significant role in this complication [5]. In 2013 we reported a muscle union procedure that was a modified version of the Jensen procedure [6]. In our procedure, the vertical muscles and the paralytic horizontal rectus muscle are joined with simple sutures without splitting the muscles [7]. This muscle union procedure effectively resolved ocular deviation and diplopia in the five patients examined in our pilot study. Since September 2010, all patients with paralytic strabismus who required muscle transposition surgery underwent this muscle union procedure at our institution.

The aim of this study was to report our experience in performing this muscle union procedure in patients with paralytic strabismus.

## Methods

This study is a retrospective review of patients with paralytic strabismus who underwent our muscle union procedure (the details of which were reported previously) [7] between September 2010 and November 2014. We reviewed the medical records of all patients to determine their postoperative ocular deviations, surgical success rates, reoperation rates, and complications. This study was approved by the Institutional Review Board of Samsung Medical Center. All surgical methods used in this study were approved by the Institutional Review Board of Samsung Medical Center and written informed consent was obtained from each patient or his/her legal guardian before enrollment. All research was performed in compliance with the tenets of the Declaration of Helsinki. The individual described in this manuscript has given written informed consent (as outlined in the PLOS consent form) to publish these case details.

At the initial visit, all patients underwent a full ophthalmologic assessment, including visual acuity testing, refraction, evaluation of ocular alignment status, slit-lamp biomicroscopy, and fundoscopic examination. Ideally, ocular alignment measurements were obtained using prism alternate cover testing at 6-m fixation and 33-cm fixation; however, when this was not possible, the Krimsky test was performed at 33-cm fixation. For fixation, a 6/9 visual acuity symbol was employed. Voluntary ductions were performed on all patients using a 4-point scale ranging from 0 to -4 as follows: 0 = patient has full movement in both eyes; -2 = patient is unable to move the affected eye past the midline; -3 = patient is unable to move the affected eye to the midline; and -4 = patient has no adducting movement in the affected eye. Two examiners (S.O. and K.P.) performed all ocular alignment tests and surgeries.

**Fig 1:**
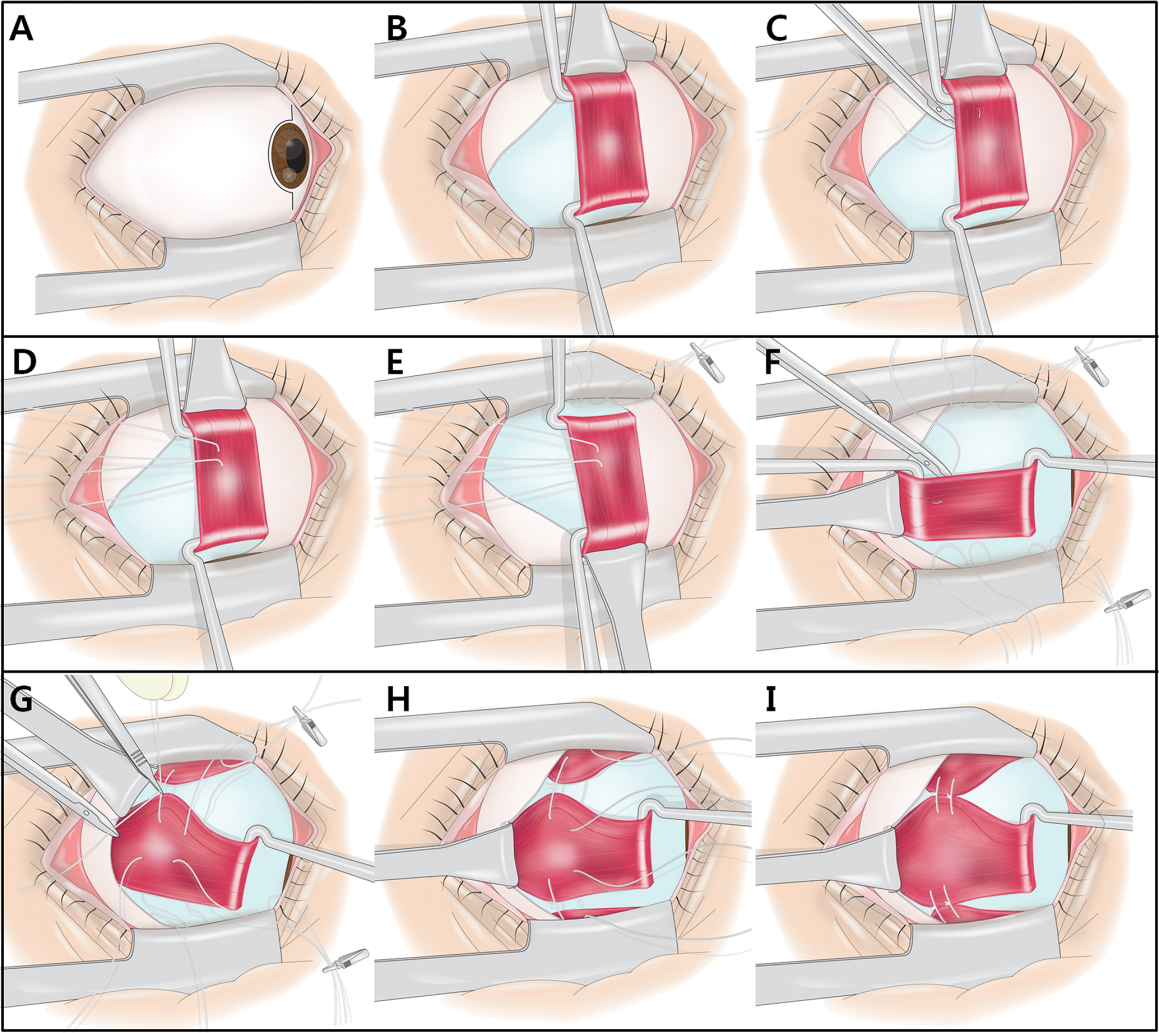
Example of the muscle union procedure in a patient with a sixth cranial nerve palsy. (A) The muscles were approached using an approximately 160-degree limbal incision. (B) The superior rectus muscle was isolated. (C, D) A number of 5–0 nonabsorbable sutures were passed through approximately 1/3 to 1/2 of the width of the muscle belly at positions 8 mm and 6 mm posterior to the muscle insertions, without muscle splitting. (E) The inferior rectus muscle was isolated, and a number of 5–0 nonabsorbable sutures were placed in the same manner as in the superior rectus muscle. (F) The 5–0 nonabsorbable sutures that had been previously placed in the vertical rectus muscles were placed at positions 8 mm and 6 mm posterior to the muscle insertions of the lateral rectus muscle. (G, H) The first set of sutures at 8 mm posterior to the insertion was loosely tied to approximate the vertical rectus muscles near the lateral rectus muscle. Special care was taken to prevent unbalanced lateral rectus muscle displacement during the first tie. (I) The second set of sutures was tied 6 mm posterior to the insertion. Surgical assistants helped by pulling the vertical rectus muscles using forceps to ensure tight ties.

In the muscle union procedure, a suture was used to approximate the vertical rectus muscles and the paralytic horizontal rectus muscle (Fig 1). The muscles were approached using an approximately 160-degree limbal incision. The superior rectus, inferior rectus, and paralytic horizontal rectus muscles were isolated separately. Care was taken to free the frenulum between the superior rectus and superior oblique muscles [8] and to free the capsulopalpebral lower lid retractors from the inferior rectus muscle. Approximately 1/3 to 1/2 of each muscle belly was sutured between the paralytic horizontal rectus and superior rectus muscles and between the paralytic horizontal rectus and inferior rectus muscles. A 5–0 nonabsorbable polyester suture was placed 8 mm posterior to the muscle insertion without splitting the muscle (Fig 1). The first suture was tied loosely to approximate the vertical rectus muscles near the paralytic horizontal rectus muscle. Special care was taken to leave at least one intact anterior ciliary artery in each of the nasal vertical muscle stumps. Special care was taken to prevent unbalanced horizontal rectus muscle displacement during placement of the first suture. The second suture was placed 6 mm posterior to the insertion. A tight suture was placed adjacent to the paralytic horizontal rectus with the help of surgical assistants who pulled the vertical rectus muscles using forceps. In two patients who had undergone partial tendon transposition and augmentation with intermuscular suture, the previously transposed vertical rectus muscles were dissected and sutured between the paralytic horizontal rectus and the previously transposed vertical rectus muscles. They were also sutured between the previously transposed vertical rectus muscles and nontransposed vertical rectus muscles at points 10 and 8 mm posterior to their insertion, respectively.

Recession surgery of the antagonist muscle was performed when the antagonist muscle was tight on the forced duction test during the operation, with the exception of one patient who had already undergone antagonist muscle recession surgery.

Postoperative assessment was performed 1 day, 1 week, 2 months, 6 months, and 12 months after surgery and then every year thereafter. Postoperative examinations were performed in the same manner as the preoperative examinations. Postoperative alignment was considered satisfactory if the deviation was 10 prism diopters (PD) or less. Overcorrection and undercorrection were defined as deviations of more than 10 PD in the appropriate directions. Base-out prism glasses were prescribed to patients with persistent diplopia associated with postoperative residual deviation 10 PD or less at 2 months postoperation. Reoperation was recommended if constant ocular deviation of more than 10 PD persisted for 6 months after surgery and the patient had significant double vision or cosmetic concerns.

## Results

**Fig 2:**
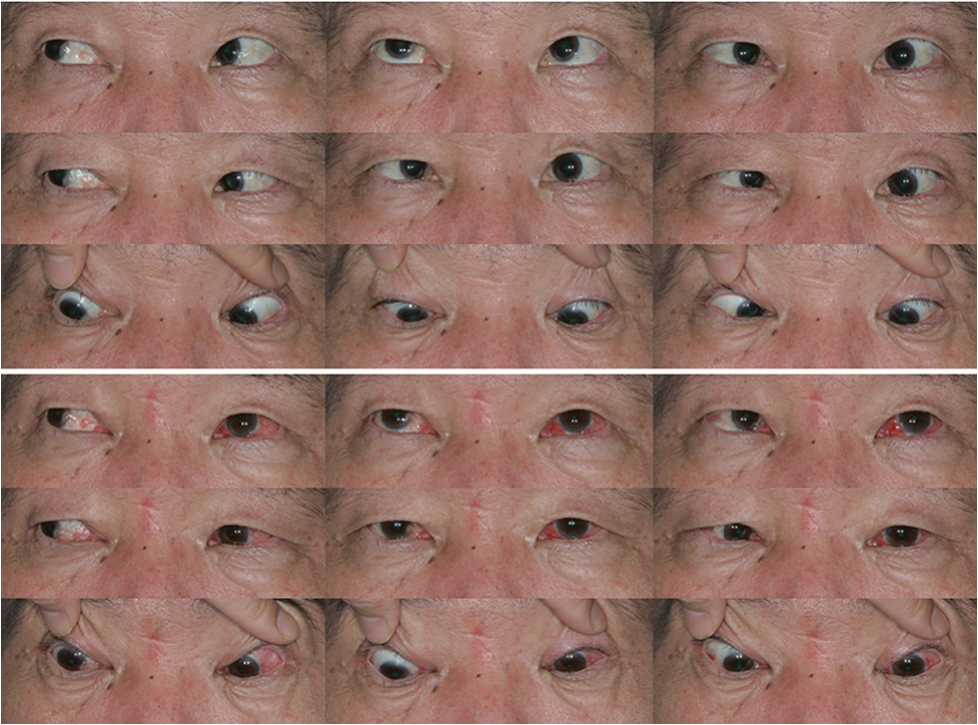
A 56-year-old man with an acquired left sixth nerve palsy due to a brain tumor. Before (top) and 1 month after (bottom) the muscle union procedure and medial rectus recession of 6 mm in the left eye.

**Fig 3:**
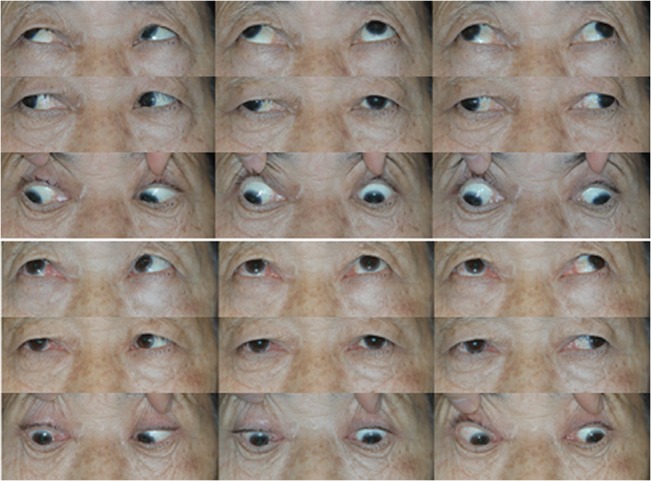
A 61-year-old man with a medial rectus palsy after sinus surgery. Before (top) and 1 week after (bottom) the muscle union procedure and lateral rectus recession of 10mm in the left eye.

We identified 27 patients with paralytic strabismus who underwent our muscle union procedure. Seventeen patients had a sixth cranial nerve palsy, seven patients had a third cranial nerve palsy, and three patients had a medial rectus muscle palsy after endoscopic sinus surgery. A summary of the relevant patient characteristics is provided in Table 1. The mean patient age was 40 ± 18 years (range, 7 to 75 years). Etiologies included brain tumor (n = 9), congenital cranial nerve palsy (n = 6), trauma (n = 5), previous sinus surgery (n = 3), intracranial hemorrhage (n = 2), ischemic brain injury (n = 1), and hydrocephalus (n = 1). The preoperative angle of horizontal deviation in the primary position was 56 ± 21 PD (range 20 to 100 PD). The mean follow-up duration was 12 ± 9 months (range 1 to 27 months). Nine (33%) patients had a history of previous rectus muscle surgery and 3 (11%) patients had a history of previous partial tendon transposition surgery. Rectus muscle recession was combined with the muscle union procedure in 13 (48%) patients. Fourteen (52%) patients had diplopia in the primary position and six (22%) patients had abnormal head posture preoperatively. Representative cases are shown in Figs 2 and 3. Postoperative data regarding ocular alignment in the primary position, ocular motility, and reoperation rates are shown in Table 2. The mean final postoperative angle of horizontal deviation in the primary position was 8 ± 13 PD (range, 0 to 45 PD). The success rate was 74% at the final visit. No reoperations were required in the follow-up period; however, recession surgery was planned for one patient with a third cranial nerve palsy whose residual deviation was 40 PD. Diplopia in the primary position was resolved in 12 (86%) patients.

**Table 1:** Preoperative characteristics of patients with paralytic strabismus who underwent the muscle union procedure (n = 27).

Characteristic	
Type of paralysis (n)	
Third cranial nerve palsy	17 (63%)
Sixth cranial nerve palsy	7 (26%)
Medial rectus muscle palsy after sinus surgery	3 (11%)
Age at the time of surgery (mean ± SD)	40 ± 18 years
Duration of paralytic strabismus (mean ± SD)	12 ± 18 years
Pre-op horizontal deviation	56 ± 21 PD
Pre-op vertical deviation (mean ± SD)	6 ± 9 PD
Pre-op limitation of the paralytic horizontal extraocular muscle* (mean ± SD)	-3.5 ± 0.7
Mean follow-up duration after the surgery (mean ± SD)	12 ± 9 months
Previous history of strabismus surgery (n)	
Antagonist muscle recession (%)	2 (7%)
Antagonist muscle recession & paralytic muscle resection (%)	5 (19%)
Partial tendon transposition and antagonist muscle recession (%)	3 (11%)
Total	10 (37%)

SD = standard deviation; pre-op = preoperative; PD = prism diopters.

*Voluntary ductions were performed using a 4-point scale ranging from 0 to -4: 0 = patient has full movement; -3 = patient is unable to move the affected eye past the midline; and -4 = patient is unable to move the affected eye to the midline.

**Table 2:** Preoperative and postoperative data of the patients who underwent the muscle union procedure for paralytic strabismus.

	Sixth nerve palsy (n = 17)	Third nerve palsy (n = 7	Medial rectus palsy after sinus surgery (n = 3)	Total (n = 27)
**Alignment 1 week after surgery (n = 27)**				
Successful alignment (%)*	14 (82%)	3 (43%)	2 (67%)	19 (70%)
Under-correction (%)^†^	0 (0%)	4 (57%)	1 (33%)	5 (19%)
Overcorrection (%)^‡^	3 (18%)	0 (0%)	0 (0%)	3 (11%)
Change in horizontal deviation (Mean±SD)^a^	59±25 PD	48±21 PD	64±5 PD	56±23 PD
Change in limitation of paralytic muscle (Mean±SD)^b^	1.3±1.0	1.0±1.0	1.3±1.2	1.2±1.0
**Alignment 2 months after surgery (n = 25)**				
Successful alignment (%)*	14 (93%)	3 (43%)	2 (67%)	19 (76%)
Under-correction (%)^†^	1 (7%)	4 (57%)	1 (33%)	6 (24%)
Overcorrection (%)^‡^	0 (0%)	0 (0%)	0 (0%)	0 (0%)
Change in horizontal deviation (Mean±SD)^a^	52±21 PD	40±15 PD	62±9 PD	50±19 PD
Change in limitation of paralytic muscle (Mean±SD)^b^	1.3±0.9	1.4±1.3	1.3±1.2	1.4±1.0
**Alignment 6 months after surgery (n = 21)**				
Successful alignment (%)*	14 (100%)	1 (25%)	2 (67%)	17 (81%)
Under-correction (%)^†^	0 (0%)	3 (75%)	1 (33%)	4 (19%)
Overcorrection (%)^‡^	0 (0%)	0 (0%)	0 (0%)	0 (0%)
Change in horizontal deviation (Mean±SD)^a^	52±22 PD	34±10 PD	65±4 PD	50±21 PD
Change in limitation of paralytic muscle (Mean±SD)^b^	1.4±0.9	1.75±1.7	1.7±1.5	1.5±1.1
**Reoperation (%)**	0 (0%)	0 (0%)	0 (0%)	0 (0%)

SD = standard deviation; PD = prism diopters.

*Successful alignment was defined as a deviation ≤10 prism diopters.

^†^Under-correction was defined as a residual deviation more than 10 PD.

^‡^Overcorrection was defined as an overcorrected deviation more than 10 PD.

^a^Change in horizontal deviation between postoperative deviation and preoperative deviation.

^b^Change in the limitation of the horizontal paralytic extraocular muscle between postoperative and preoperative deviation.

Voluntary ductions were performed using a 4-point scale ranging from 0 to -4: 0 = patient has full movement in the affected eye; -2 = patient is unable to move the affected eye past the midline; -3 = patient is unable to move the affected eye to the midline; and -4 = patient has no adducting movement in the affected eye.

In the patients with a sixth cranial nerve palsy, the mean preoperative horizontal angle of deviation in the primary position was 53 ± 22 PD, compared with 1 ± 3 PD at the final postoperative visit. At this visit, 13 patients were orthophoric, 1 patient had an exophoria of 4 PD, and 3 patients had esotropias of 6 to 10 PD in the primary position. One patient who lost his vision in the fellow eye due to preoperative trauma had a newly developed vertical deviation of 6 PD. However, since the vertical deviation posed no significant cosmetic concerns, no further treatment was recommended. Two patients with residual esotropias less than or equal to 10 PD did not have double vision due to suppression of the esotropic eye, which persisted after the preoperative exam. The other patient with a residual deviation of 10 PD was prescribed prism glasses to resolve his diplopia. In patients with a third cranial nerve palsy, the mean preoperative horizontal angle of deviation in the primary position was 58 ± 23 PD, compared with 24 ± 15 prism diopters at the final postoperative visit. At this visit, 2 patients had an exotropia of 10 PD or less, 2 patients had an exotropia of 15 PD, and 3 patients had an exotropia of 35 to 45 PD in the primary position. The mean preoperative vertical angle of deviation in the primary position was 12 ± 12 PD, compared with 4 ± 4 PD at the final postoperative visit. No patient exhibited a postoperative increase in vertical deviation; similarly, no patient exhibited double vision due to suppression of the exotropic eye which had persisted since the preoperative exam. The mean preoperative vertical angle of deviation in the primary position was 12 ± 12 PD, compared with 4 ± 4 PD at the final postoperative visit. No postoperative increases in vertical deviation were observed.

In the patients with a medial rectus palsy after sinus surgery, the mean preoperative angle of deviation in the primary position was 61 ± 1 PD, compared with 12 ± 11 PD at the final postoperative visit. At this visit, 1 patient was orthophoric, 1 patient had an exotropia of 12 PD, and the other patient had an exotropia of 22.5 PD. Only one patient had a preoperative vertical deviation of 15 PD that persisted after the operation. One patient who was orthophoric after the operation had resolved double vision, but another patient with a residual exotropia of 12 PD and a vertical deviation of 15 PD had persistent double vision. The other patient with an exotropia of 22.5 PD did not have double vision due to decreased vision in the exotropic eye, which had persisted since the preoperative exam.

No intraoperative surgical complications or cases of anterior segment ischemia were reported. Two patients developed a transient mild increase in the intraocular pressure of the operative eye on postoperative day one, both cases of which resolved within 1 week.

## Discussion

The goal of our muscle union procedure is to obtain maximum horizontal vector from the vertical rectus muscles while limiting stress on the sclera caused by suture fixation. Uniting the vertical muscles with the horizontal rectus muscle changes the direction of the force vectors of the vertical rectus muscles toward the action direction of the lateral rectus muscle. In addition, this union displaces the vertical rectus muscles more than conventional transposition surgeries do. Since the vertical rectus muscles exhibit a constant stiffness, the horizontal force increases according to Hook’s law, which states that the force needed to extend or compress a spring by a given distance is proportional to that distance, i.e. *F* = *kX* (*F* = force, *k* = stiffness, *X* = displacement). Since the muscle union procedure does not require muscle splitting or dissection of the vertical muscles from their insertions, this method causes less bleeding and is quicker to perform than other types of transposition surgeries. This technique could also potentially minimize the risk of anterior segment ischemia.

Based on the results of the final visit, 74% of all patients who underwent the muscle union procedure had successful surgery. Our muscle union procedure was able to resolve large degrees of horizontal strabismus, even in cases of strabismus fixus. We propose that the reason that our muscle union procedure could correct large degrees of horizontal deviation in patients with no force is the increased vector force resulting from the increased muscle displacement.

The success rate was highest in patients with a sixth cranial nerve palsy (up to 100%) and lowest in patients with a third cranial nerve palsy (25%). We hypothesized that the transposition surgery is less effective in third cranial nerve palsies compared with sixth cranial nerve palsies because the vertical rectus muscles might be involved in the former.

A previous study of transposition surgery reported vertical deviation in up to 20% of all patients [9]. We noted 1 patient who experienced vertical deviation after the muscle union procedure. This patient developed vertical deviation 2 years after the surgery, was blind in the fellow eye, and did not have fusion ability. An unbalanced vector force can induce vertical deviation in patients who undergo our muscle union procedure. After examining the patient who exhibited postoperative vertical deviation, we took special care to perform balanced union procedures.

Regarding complications, no cases of anterior segment ischemia were recorded. Two cases of transient increases in intraocular pressure were noted. Since the intraocular pressure increased immediately after the operation and then continuously decreased to a normal level before discontinuation of steroid eye drops in these cases, we propose that the increases may have been caused by mechanical pressure from tightened muscles. In both cases, intraocular pressure normalized within one week without any treatment.

It may be possible to modify the effect of the surgery on the deviation by changing the location of the muscle union, which would potentially broaden the surgical indication of this procedure. Further studies are needed to investigate this possibility.

This study did have several limitations. First, the sample size was small; thus, a large scale prospective study is needed to confirm the safety and efficacy of our muscle union procedure. Second, the follow-up period was relatively short. However, the final outcomes were very similar to those seen after 6 months in several patients for whom long term follow-up data are available. Thus, we infer that any postoperative changes would be minimal by 6 months after the operation. Third, no control group was included. Finally, the postoperative results could have been subject to observational bias.

In conclusion, our muscle union procedure was effective in patients with paralytic strabismus, especially when the patient had a large angle of deviation. This muscle union procedure is thus a suitable option for transposing the muscles in patients with paralytic strabismus, especially those with large-angle deviation or a significant residual angle after conventional surgery.
